# Enhancing the recovery of a temporal sequence of images using joint deconvolution

**DOI:** 10.1038/s41598-018-22811-x

**Published:** 2018-03-27

**Authors:** Piergiorgio Caramazza, Kali Wilson, Genevieve Gariepy, Jonathan Leach, Stephen McLaughlin, Daniele Faccio, Yoann Altmann

**Affiliations:** 1 Institute of Photonics and Quantum Sciences, School of Engineering & Physical Sciences, Heriot-Watt University, EH14 4AS Edinburgh, United Kingdom; 2 Institute of Sensors, Signals and Systems, School of Engineering & Physical Sciences, Heriot-Watt University, EH14 4AS Edinburgh, United Kingdom

## Abstract

In this work, we address the reconstruction of spatial patterns that are encoded in light fields associated with a series of light pulses emitted by a laser source and imaged using photon-counting cameras, with an intrinsic response significantly longer than the pulse delay. Adopting a Bayesian approach, we propose and demonstrate experimentally a novel joint temporal deconvolution algorithm taking advantage of the fact that single pulses are observed simultaneously by different pixels. Using an intensified CCD camera with a 1000-ps gate, stepped with 10-ps increments, we show the ability to resolve images that are separated by a 10-ps delay, four time better compared to standard deconvolution techniques.

## Introduction

Recent development of high-temporal resolution cameras, such as intensified charge-coupled device (ICCD), single-photon avalanche diode (SPAD) and streak cameras, have allowed the study of picosecond phenomena by means of direct measurements. Such high-speed cameras have paved to the way to a wide range of applications, including direct observation of dynamical light phenomena, such as laser-induced plasma^1^, imaging around corners by means of the laser echoes^2–4^ and real-time fluorescence lifetime imaging^5^. Although state-of-the art single-photon detectors are now able to record photon time-of-arrivals with picosecond resolution, the intrinsic system limitations (e.g. the camera efficiency and temporal response) and signal noise/dephasing induced by propagation through complex media require the development of novel computational methods adapted to the discrete and sparse nature of the recorded data (single-photon detection events) in order to efficiently extract information about the phenomena of interest. Moreover, there is growing interest in exploiting spatial information to improve temporal resolution. In particular, the assumption of space-sparsity has been used extensively in order to improve the spatial deconvolution of images for problems like deblurring^6^ and denoising^7,8^. Moreover, recent research has shown a renewed interest for free space communication systems and in particular systems that use also the spatial dimension to encode information, e.g. through a judicious choice of the spatial beam modes^9,10^.

In this work, we take advantage of the spatial dimensionality offered by high-speed cameras in order to provide a new temporal deconvolution method with increased temporal resolution. Our algorithm takes advantage of the fact that within a given frame, groups of pixels may share common temporal information, independent of any spatial correlation. This is typically the case when, for example, different spatial patterns are encoded in a series of light pulses: each laser pulse contains information that is detected across several pixels in the receiving camera. Therefore, we build a joint deconvolution method, which solves the deconvolution problem by processing jointly groups of pixels, i.e., by regularising the pixel intensity values on a frame by frame basis. The ability to penalise individual temporal frames without affecting neighbouring frames results in a significant improvement of our ability to discriminate events that are temporally close, when compared to state-of-the-art deconvolution techniques based on Poisson noise assumptions and using established convex optimisation techniques^11,12^. In our experiments, we employ an ICCD camera with a 1000-ps gate that is stepped in 10-ps increments. Experimentally, we are able to distinguish two images encoded in two separate laser pulses that are delayed by 10-ps, with a temporal resolution that is two orders of magnitude better than the width of the gate, corresponding to an improvement by a factor of four in the temporal resolution with respect to standard techniques. Although the proposed computational method requires the signal from several pixels, it does not rely on specific spatial correlations between adjacent pixels. It is thus broadly applicable to imaging applications for which the observed intensity fields can present significant intensity variations among neighbouring pixels.

## Method

### collaborative sparse reconstruction

Deconvolution problems are usually solved by minimising an appropriate cost function. Such cost functions typically include a data fidelity term which quantifies the similarity between the observed data **y** and its approximation, which results from the analytical convolution of the original signal of interest **x** by the response function of the sensing or imaging system. The convolution process is often considered as a linear process (denoted as **F** here), and the convolved signal is **Fx**. The original signal is recovered by minimising the cost function with respect to **x**, and possibly other unknowns such as background illumination. A typical example, which also serves to showcase the novelty of our joint deconvolution method, is the SPIRAL algorithm^12^. The SPIRAL algorithm can be applied to restore images corrupted by a linear operator (e.g., a blurring or down-sampling operator) in the presence of Poisson noise, but for the purpose of this paper we will introduce its principle for time series restoration. Consider the cost function.

1 $${C}_{{{\bf{y}}}_{n}}({{\bf{x}}}_{n},{b}_{n},\lambda ,{\lambda }_{b})={C}_{1}({{\bf{y}}}_{n},{\bf{F}}{{\bf{x}}}_{n}+{{\bf{b}}}_{n})+\lambda {\varphi }_{x}({{\bf{x}}}_{n})+{\lambda }_{b}{\varphi }_{b}({b}_{n}))+{1}_{{{\mathbb{R}}}^{+}}({{\bf{x}}}_{n},{b}_{n}),$$

where ${{\bf{y}}}_{n}={[{y}_{n\mathrm{,1}},\ldots ,{y}_{n,T}]}^{T}$ is the discrete time series of length *T*, recorded in a given pixel *n*, whose sampling rate is determined by the physical specifications of the detector, where ${{\bf{x}}}_{n}={[{x}_{n\mathrm{,1}},\ldots ,{x}_{n,{T}_{x}}]}^{T}$ is the original signal to be recovered. Note that the data sampling rate is not required to match the sampling rate of **x**_*n*_, which can be higher than that imposed by the detector (i.e., when *T*_*x*_ > *T*). In Eq. (1), **b**_*n*_ is a constant vector combining the ambient illumination and detector dark count level, which are assumed to be constant over time, but can vary across pixels. Thus, the elements of **b**_*n*_ are all equal to the same value *b*_*n*_ > 0. The cost function *C*_1_(·) corresponds to the data fidelity term and depends on the underlying observation noise model. For applications where the Gaussian noise assumption holds, the data fidelity term becomes

2 $${C}_{1}({{\bf{y}}}_{n},{\bf{F}}{{\bf{x}}}_{n}+{{\bf{b}}}_{n})=||{{\bf{y}}}_{n}-({\bf{F}}{{\bf{x}}}_{n}+{{\bf{b}}}_{n}{)||}_{2}^{2},$$

where ||·||_2_ denotes the standard ${\ell }_{2}$-norm. When the Poisson noise model is more accurate, the data fidelity term derived from the data negative log-likelihood becomes

3 $${C}_{1}({{\bf{y}}}_{n},{\bf{F}}{{\bf{x}}}_{n}+{{\bf{b}}}_{n})=\sum _{t=1}^{T}[{z}_{n,t}-{y}_{n,t}\,\mathrm{log}({z}_{n,t})],$$

where ${{\bf{z}}}_{n}={[{z}_{n\mathrm{,1}},\ldots ,{z}_{n,T}]}^{T}={\bf{F}}{{\bf{x}}}_{n}+{{\bf{b}}}_{n}$. The second and third terms are regularisation terms which encode our prior knowledge about the unknown signal to be recovered and the background illumination, respectively. The influence of these terms is controlled by two positive parameters *λ* and *λ*_*b*_ (the larger the values of these parameters, the more significant the impact of the corresponding regularisation on the recovered signal). Lastly, the fourth term on the right-hand side of (1) is an indicator defined on ${{\mathbb{R}}}^{+}$ to ensure the positivity of (**x**_*n*_, *b*_*n*_).

Among the various regularisations that can be used, convex (with respect to **x**_*n*_) sparsity promoting functions are particularly relevant here since we expect to recover a reduced number of pulses, relatively short compared to the sampling rate of **x**_*n*_. Moreover, they allow state-of-the art convex optimization techniques to be used to recover **x**_*n*_. A classical approach consists of adopting an ${\ell }_{1}$ regularisation, i.e., setting $\varphi ({{\bf{x}}}_{n})=||{{\bf{x}}}_{n}{||}_{1}={\sum }_{t}|{x}_{n,t}|$, which will force in a similar fashion all the elements of **x**_*n*_ to be small. Although generally efficient, the ${\ell }_{1}$ regularisation does not lead to sufficiently sparse solutions to solve our deconvolution problem satisfactorily, as will be seen in the next section. In this work, we thus adopt a so-called weighted ${\ell }_{1}$ regularisation such that the second term in Eq. (1) becomes ${\sum }_{t}\,{\lambda }_{t}|{x}_{n,t}|$, where the parameters ${\lambda }_{t}$ are now allowed to vary in time. This allows locally for larger values for ${x}_{n,t}$ when ${\lambda }_{t} > 0$ is small, when compared to the standard ${\ell }_{1}$ regularisation. Although more flexible, the weighted ${\ell }_{1}$ approach requires the selection of ${T}_{x}$ additional parameters (a single $\lambda$ is required for the original ${\ell }_{1}$-based regularisation in Eq. (1)), whose values have a significant impact on the solution and which are difficult to adjust in practice, especially when information is analysed one individual pixel at a time.

We therefore propose a joint deconvolution method where we simultaneously process groups of pixels i.e., the entire ICCD camera array described in the next section, in order to reduce estimation uncertainty (as pixels are expected to contain redundant information). This allows us to estimate the ${T}_{x}$ additional parameters $\{{\lambda }_{t}\}$, which are assumed to be shared across all the pixels of the group. In other words, we expect some frames to consist on average of high intensities while other frames are expected to be darker on average. Assuming that we observe a set of *N* pixels over time, the cost function in Eq. (1) becomes

4 $${C}_{{\bf{Y}}}({\bf{X}},{\bf{b}},{\boldsymbol{\lambda }})=\sum _{n}{C}_{1}({{\bf{y}}}_{n},{\bf{F}}{{\bf{x}}}_{n}+{{\bf{b}}}_{n})+\sum _{n,t}{\lambda }_{t}|{x}_{n,t}|+{\lambda }_{b}\sum _{n}|{b}_{n}|+\psi ({\boldsymbol{\lambda }})+{1}_{{{\mathbb{R}}}^{+}}({\bf{X}},{\bf{b}},{\boldsymbol{\lambda }}),$$

where ${\bf{Y}}=[{{\bf{y}}}_{1},\ldots ,{{\bf{y}}}_{N}]$, ${\bf{X}}=[{{\bf{x}}}_{1},\ldots ,{{\bf{x}}}_{N}]$, ${\bf{b}}=[{b}_{1},\ldots ,{b}_{N}]$ and ${\boldsymbol{\lambda }}={[{\lambda }_{1},\ldots ,{\lambda }_{Tx},{\lambda }_{b}]}^{T}$. Note that in Eq. (4), we introduce an additional regularisation term $\psi ({\boldsymbol{\lambda }})$ to encode prior information (e.g. positivity constraints) available about ${\boldsymbol{\lambda }}$. This term will be further discussed later in this section. As mentioned above, estimating **X** by minimising the cost function in (4) can significantly improve signal restoration performance, provided that ***λ*** is properly adjusted. Unfortunately, the cost function in Eq. (4) is in general highly multimodal, and thus particularly difficult to minimise globally with respect to $({\bf{X}},{\bf{b}},{\boldsymbol{\lambda }})$ using optimisation techniques. However, this cost function (up to an additive constant) can be interpreted in a statistical framework as the negative logarithm of the joint probability density function of $({\bf{X}},{\bf{b}},{\boldsymbol{\lambda }})$, given the observed data **Y**, denoted as $f({\bf{X}},{\bf{b}},{\boldsymbol{\lambda }}|{\bf{Y}})$; that is

5 $$\exp [-{C}_{{\bf{Y}}}({\bf{X}},{\bf{b}},{\boldsymbol{\lambda }})]\propto f({\bf{X}},{\bf{b}},{\boldsymbol{\lambda }}|{\bf{Y}}\mathrm{).}$$

This property is particularly interesting as it enables a larger range of statistical tools to satisfactorily solve the deconvolution problem. Note that for Eq. (5) to apply, $\psi ({\boldsymbol{\lambda }})$ should correspond to a valid prior distribution for ${\boldsymbol{\lambda }}$. Here, $\psi ({\boldsymbol{\lambda }})$ is defined such that the resulting prior distribution $f({\boldsymbol{\lambda }})$ consists of *T*_*x*_ independent gamma distributions. These prior distributions are set to be weakly informative so that they do not bias unnecessarily the intensity estimation while defining a proper hierarchical Bayesian model.

Instead of minimising ${C}_{{\bf{Y}}}({\bf{X}},{\bf{b}},{\boldsymbol{\lambda }})$ which is computationally challenging and which corresponds to estimating $({\bf{X}},{\bf{b}},{\boldsymbol{\lambda }})$ via *maximum a posteriori* estimation, here we resort to a Markov Chain Monte Carlo (MCMC) method^13^ to approximate the *marginal posterior means* of interest

6 $$\hat{{\bf{X}}}={\langle {\bf{X}}\rangle }_{{\bf{Y}}},{\rm{and}}\,\hat{{\boldsymbol{\lambda }}}={\langle {\boldsymbol{\lambda }}\rangle }_{{\bf{Y}}}\mathrm{.}$$

The main goal the MCMC method used in this work is to generate random variables distributed according to $f({\bf{X}},{\bf{b}},{\boldsymbol{\lambda }}|{\bf{Y}})$ and to use the generated samples to approximate numerically the high-dimensional integrals involved in the computation of the expectations in Eq. (6). Precisely, in order to generate random variables asymptotically distributed according to $f({\bf{X}},{\bf{b}},{\boldsymbol{\lambda }}|{\bf{Y}})$, we resort to a Metropolis-within Gibbs sampler to sample sequentially and iteratively according to the conditional distributions $f({\bf{X}},{\bf{b}}|{\bf{Y}},{\boldsymbol{\lambda }})$ and $f({\boldsymbol{\lambda }}|{\bf{Y}},{\bf{X}},{\bf{b}})$. While the conditional distribution $f({\boldsymbol{\lambda }}|{\bf{Y}},{\bf{X}},{\bf{b}})$ reduces to a product of independent gamma distributions, which is easy to sample from, the conditional distribution $f({\bf{X}},{\bf{b}}|{\bf{Y}},{\boldsymbol{\lambda }})$ is a non-standard distribution and accept-reject procedures are required to update $({\bf{X}},{\bf{b}})$. Due to large dimensionality of $({\bf{X}},{\bf{b}})$ and the high correlation between these variables, we resort to constrained Hamiltonian Monte Carlo (HMC) updates which use the local curvature of the distribution $f({\bf{X}},{\bf{b}}|{\bf{Y}},{\boldsymbol{\lambda }})$ to propose candidates in regions of high probability. This approach allows better mixing properties than more standard random walk alternative strategies. The pseudo code of the proposed sampler is detailed below. The marginal posterior means $\hat{{\bf{X}}}$ and $\hat{{\boldsymbol{\lambda }}}$ are approximated by averaging the generated variables after having removed the first ${N}_{{\rm{bi}}}$ iterations of the sampler which correspond to the burn-in period of the sampler. The duration of this transient period and the total number of iterations ${N}_{{\rm{MC}}}$ are set by visual inspection of the chains from preliminary runs.

**Algorithm 1:**
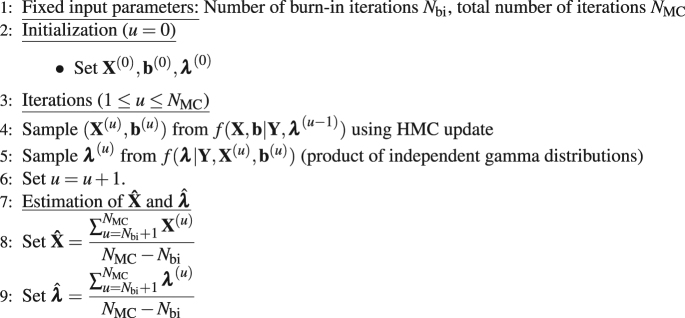
Proposed MCMC method.

For the deconvolution problem addressed in this paper, it turns out that in practice, the proposed method is particularly efficient both in terms of convergence speed and reliability of the estimated intensity profiles. It is also important to recall that this method is fully unsupervised in the sense that it does not require the user to tune regularisation parameters affecting the obtained solution. The details of implementing the MCMC method can be found for example in ref.^13^.

The estimation strategy explained above exploits the natural redundancy between the $N$ pixels considered but does not explicitly rely on the presence of spatially structured intensity fields. For applications where the intensity fields are indeed structured (i.e. they contain images), we propose an additional processing step to refine the field estimated by the MCMC method. First, we threshold the regularisation parameters estimated in Eq. (6) to identify the most significant (brightest) frames within the image sequence and thus concentrate on enhancing a reduced number of significant intensities at a lower computational cost. Since the underlying intensity field is expected to consist of a reduced number of pulses, only a reduced number of frames are likely to be picked up. To perform the frame selection, we select sequentially the frame presenting the highest ${\hat{\lambda }}_{t}$ until the sum of the retained $\{{\hat{\lambda }}_{t}\}$ exceeds 99% of the sum of all the regularisation parameters. Then we introduce classical independent *total-variation* regularisations for each of the retained frames (whose indices are gathered in ${\mathscr{T}}$) to promote spatially structured intensities and minimise the following cost function

7 $${{\rm{C}}}_{{\bf{Y}},{\mathscr{T}}}({{\bf{X}}}_{{\mathscr{T}}},{\bf{b}})=\sum _{n}{C}_{1}({{\bf{y}}}_{n},\tilde{{\bf{F}}}{{\bf{x}}}_{{\mathscr{T}}}+{{\bf{b}}}_{n})+\sum _{t\in {\mathscr{T}}}\sum _{n}{\hat{\lambda }}_{t}|{x}_{n,t}|+{\hat{\lambda }}_{t}||{\bf{b}}{||}_{1}+\sum _{t\in {\mathscr{T}}}{\mu }_{t}{\rm{TV}}({{\bf{X}}}_{{\mathscr{T}},t})+{1}_{{{\mathbb{R}}}^{+}}({{\bf{X}}}_{{\mathscr{T}}},{\bf{b}})$$

where *TV*(·) denotes the total variation (TV) regularization^14^ and ${{\bf{X}}}_{{\mathscr{T}}}$ is the reduced set of frames corresponding to highest values of ${\hat{\lambda }}_{t}$ obtained from the first step. The TV-regularisation parameters have been set to ${\mu }_{t}=10{\hat{\lambda }}_{t}$. In contrast to Eq. (4), the cost function in Eq. (7) is convex with respect to $({{\bf{X}}}_{{\mathscr{T}}},{\bf{b}})$ and involves significantly fewer unknown parameters. Therefore it can be minimised efficiently using a pre-existing, state-of-the-art convex optimisation method at a low additional cost.

It is worth mentioning that the main computational cost of the proposed approach is the first step achieved using a simulation method. The proposed sampler requires a sufficiently large number of iterations to ensure reliable estimates. Although Markov chain Monte Carlo methods are usually more computationally demanding that optimization methods, the proposed method is competing with state-of-the art optimization methods as it simultaneously estimates the intensity eld and the regularization parameters. Indeed, iterative re-weighting methods are not particularly fast for this ill-posed problem. Initializing the regularization parameters too large usually leads to poor (too sparse) results while initializing with small regularization parameters induces prohibitively slow convergence rates.

### Experimental setup

**Figure 1:**
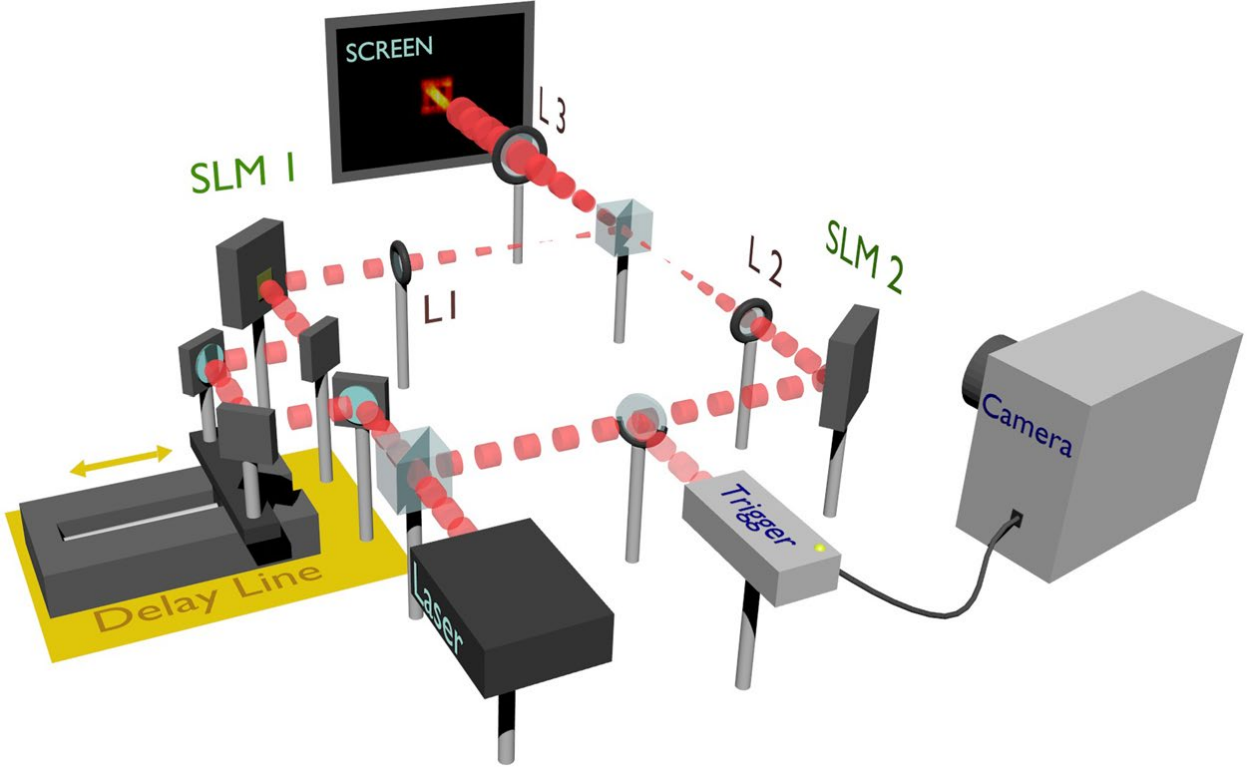
Experimental setup. A femtosecond-pulsed laser beam, repetition rate =80 MHz and wavelength *λ* = 810 nm, is used to project two spatially-overlapped, but temporally-delayed femtosecond pulses of light onto a screen. Different spatial patterns are imposed on the two pulses, an ‘N’ and a ‘mirrored Z’, by means of two SLMs. The delay is introduced by means of a variable delay line, with a range of 0–200 ps. The screen is imaged with an ICCD camera with a 1000-ps gate and 10-ps step size. An optical pulse picker is used to trigger the camera off of the femtosecond pulse.

The experimental layout is shown in Fig. 1. A femtosecond pulsed laser beam, with a repetition rate of 80 MHz, pulse width of 140 ± 20 fs, and wavelength *λ* = 810 nm, is separated by a beam-splitter into two paths, in a configuration similar to a Mach-Zehnder interferometer. Each path contains a phase spatial light modulator (SLM), which is used to tailor the spatial profile of the beam. We choose the letters ‘N’ and a ‘mirrored Z’ to obtain patterns that are significantly overlapping. Generally, the larger the overlap ratio, the harder the deconvolution. Thus, the proposed method will perform well when the images are the inverse of each other. The most challenging scenario occurs when the two images are exactly the same. In such cases, all the pixels contain either the two peaks or none and the data might not contain enough diversity to allow the algorithm to identify the actual presence of multiple peaks, in particular when their relative delay is short. A second beam-splitter then recombines the two beam paths, and the SLM planes are imaged onto a screen using lenses *L*_1_, *L*_2_ and *L*_3_ as shown in Fig. 1, so that the two spatial patterns overlap on the screen. We introduce a variable delay line into one of the paths allowing the temporal separation of the pulses to be varied from 0 ps to 200 ps. We use a standard camera lens to image the screen onto an LaVision PicoStar ICCD camera. The temporal dynamics, i.e., pulse separation, are captured using a 1000-ps gate, and stepping the gate in 10-ps increments. The camera requires an external trigger as shown in order to synchronize the acquisitions with the pulsed laser.

## Results

**Figure 2:**
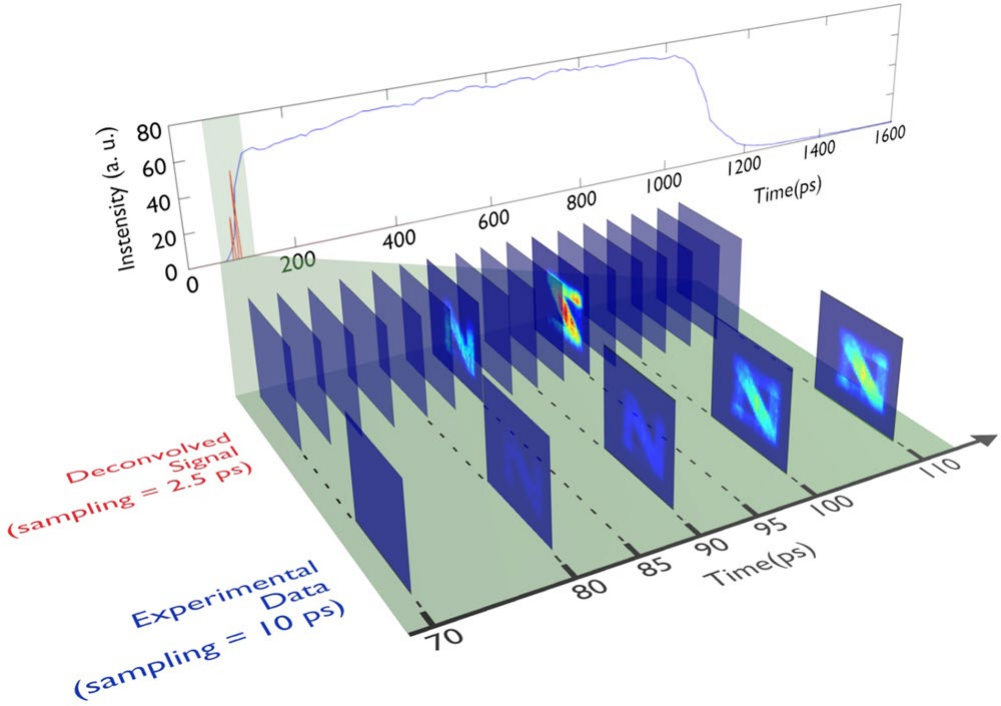
Comparison between raw data (blue line) and the deconvolved signal (red line) for two pulses separated by 10 ps with different spatial patterns, an ‘N’ and a ‘mirrored Z’. Intensity data are obtained by averaging over the image pixels frame by frame. Experimental data have been recorded employing an ICCD with a 1000-ps gate and 10-ps step size. Sampling has been improved by a factor of four for the deconvolved signal. The original signal is successfully restored with the minimum possible temporal resolution (2.5 ps, the improved sampling rate set into the retrieval algorithm).

For all the results presented in this section, the sampling period of the deconvolved signal is fixed to 2.5 ps, which corresponds to an improvement by a factor of four compared to the minimum sampling period of the observed data. First we show the capacity of our algorithm to reconstruct the signal of two pulses separated temporally by 10 ps, resulting in an improvement over the 1000-ps gate-width by two orders of magnitude. In Fig. 2 the deconvolved signal is compared with raw data. As can be seen, the algorithm is able to resolve the ill-posed problem and gives us the best possible resolution resulting in just one temporal frame for each image.

**Figure 3:**
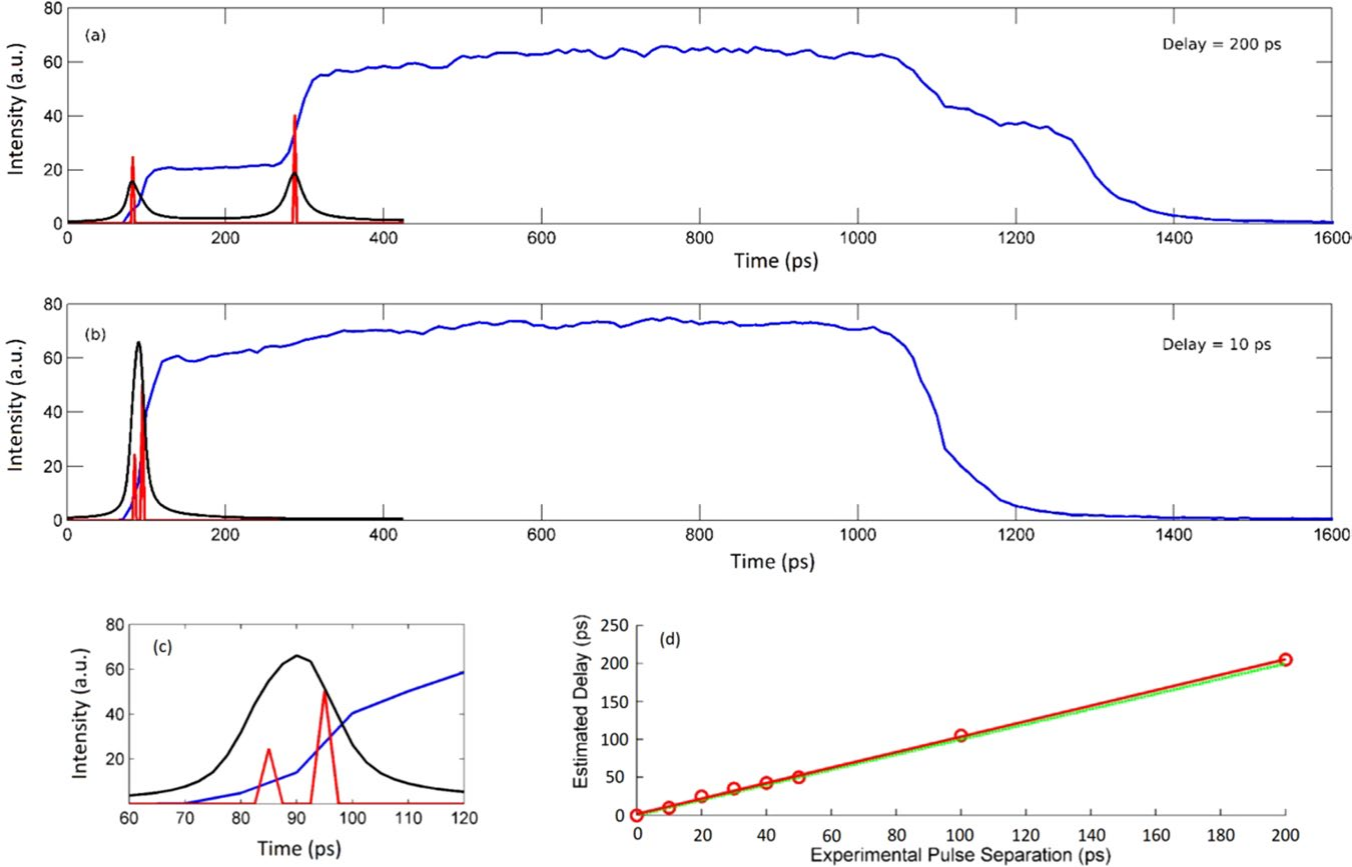
Comparison between raw data (blue line), single-pixel deconvolved signal (black line) and collaborative-method deconvolved signal (red line), for (**a**) 200 ps delay and (**b**) 10 ps delay. (**c**) zooms in on the peaks shown in (**b**). The single-pixel deconvolution, in (**a**–**c**), has been scaled by 10 × to allow for easier comparison. All the reported data are obtained by averaging over the image pixels frame by frame. Precisely, the average is calculated over the independently deconvolved different pixels’ signals. (**d**) Varying the delay between the pulses, the estimated delay calculated by our algorithm is plotted versus the expected value. Data are linearly fitted (red line) and compared with the expected trend (green dashed line).

We may then compare our joint deconvolution method with a more standard single-pixel algorithm, such as that described in equation (1). As shown in Fig. 3, the single-pixel algorithm results in peaks that are much broader than those from our joint deconvolution algorithm. For peaks delayed by 200 ps, temporal deconvolution is possible with either method. But below 40-ps delay, the single-pixel algorithm is no longer able to separate the two pulses. Conversely, the joint deconvolution method allows us to locate two pulses and therefore isolate two images that are separated by as little as 10 ps, thus resulting in a factor of four times improvement in the temporal resolution. Furthermore, we report in Fig. 3(d) the estimated delays, resulting from our algorithm by varying the imposed delay, versus the expected delay values. A good agreement is found between the linear fit of the estimated delays and the expected trend. Note that there is a difference in the amplitude of the two peaks that arises from the experimental setup (two different light paths) which attenuates more the first peak than the second peak. As can be seen in the top subplot of Fig. 3 (Fig. 3(a)), the amplitude of the second observed peak is larger (slightly less than twice larger) than the first peak. This difference can also be observed clearly in the deconvolved peaks identified by our proposed method. The conventional method provides two peaks whose maximum amplitudes are similar, however the second peak (in the right-hand side) is slightly broader than the first peak. This result arises from the fact that a single sparsity parameter is used by the standard method which tends to provides peaks with similar heights.

**Figure 4:**
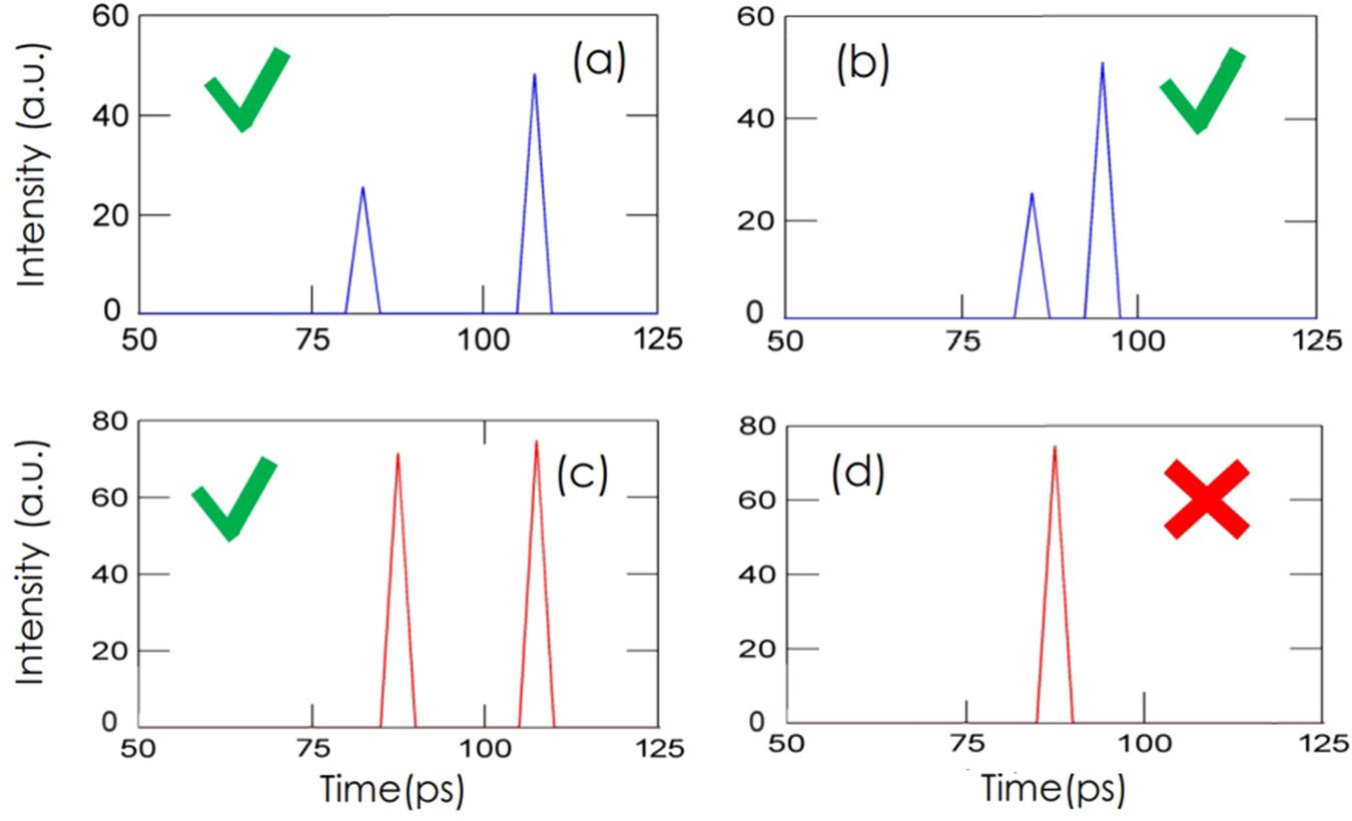
Comparison between the deconvolution’s results obtained with a step size of 10 ps (**a**,**b**) and step size of 20 ps (**c**,**d**). For (**a**,**c**) the peak separation is 20 ps, whereas for (**b**,**d**) it is 10 ps. As it can be seen in (**d**), with 20-ps step size the algorithm is no longer able to distinguish peaks separated by 10 ps.

We now briefly investigate the role of the sampling step size. In Fig. 4, we show the results of the joint deconvolution for the two different sampling step sizes, for pulses/images separated by a temporal delay of 10 ps and a delay of 20 ps. Figure 4(a,b) correspond to a step size of 10 ps and image delays of 20 ps and 10 ps respectively. Figure 4(c,d) correspond to a step size of 20 ps and delays of 20 ps and 10 ps respectively. Note that in Fig. 4(c), due to the small delay between the two pulses and the degraded sampling period (20 ps instead of 10 ps), it becomes more difficult to accurately quantify the individual amplitudes of the two peaks, which translates in more similar estimated peaks when using the proposed method. As shown in Fig. 4(d), we observe that with a 20-ps step size, the algorithm fails to restore the original signal, showing a single peak instead of two separate peaks. On the other hand, a 10-ps step size allows us to resolve two pulses with a separation of 10 ps. The failure to resolve a 10-ps delay, using a 20-ps step size suggests that the step size is one of the factors that limits the temporal resolution of our experiment. Further factors might be identified in the shape of the gate and especially in the gate’s rising time: loosely speaking, steeper rising edges provide more information and allow better resolving power from the deconvolution algorithm. We were not able to control the rise time in our experiments, so we simply highlight this point as a potential parameter to be considered when choosing a gated camera for these applications. In the experiments presented in the paper, the rise time of the gate is around 50 ps and the fall time is around 200 ps. The rise time is thus more limiting than the fall time. Moreover, sampling periods much shorter than 50 ps will have less impact on the temporal resolution than sampling periods close to or larger than the rise time of the gate. Furthermore, the method accuracy depends on the actual noise level (ambient illumination and acquisition time). Choosing a sampling period smaller for the deconvolved signal than the observed signal allows the recovery of temporal details that occur faster than the sampling period of the observed data (super-resolution), provided that the quality (e.g. noise level, sampling period) of the observed data is high enough.

**Figure 5:**
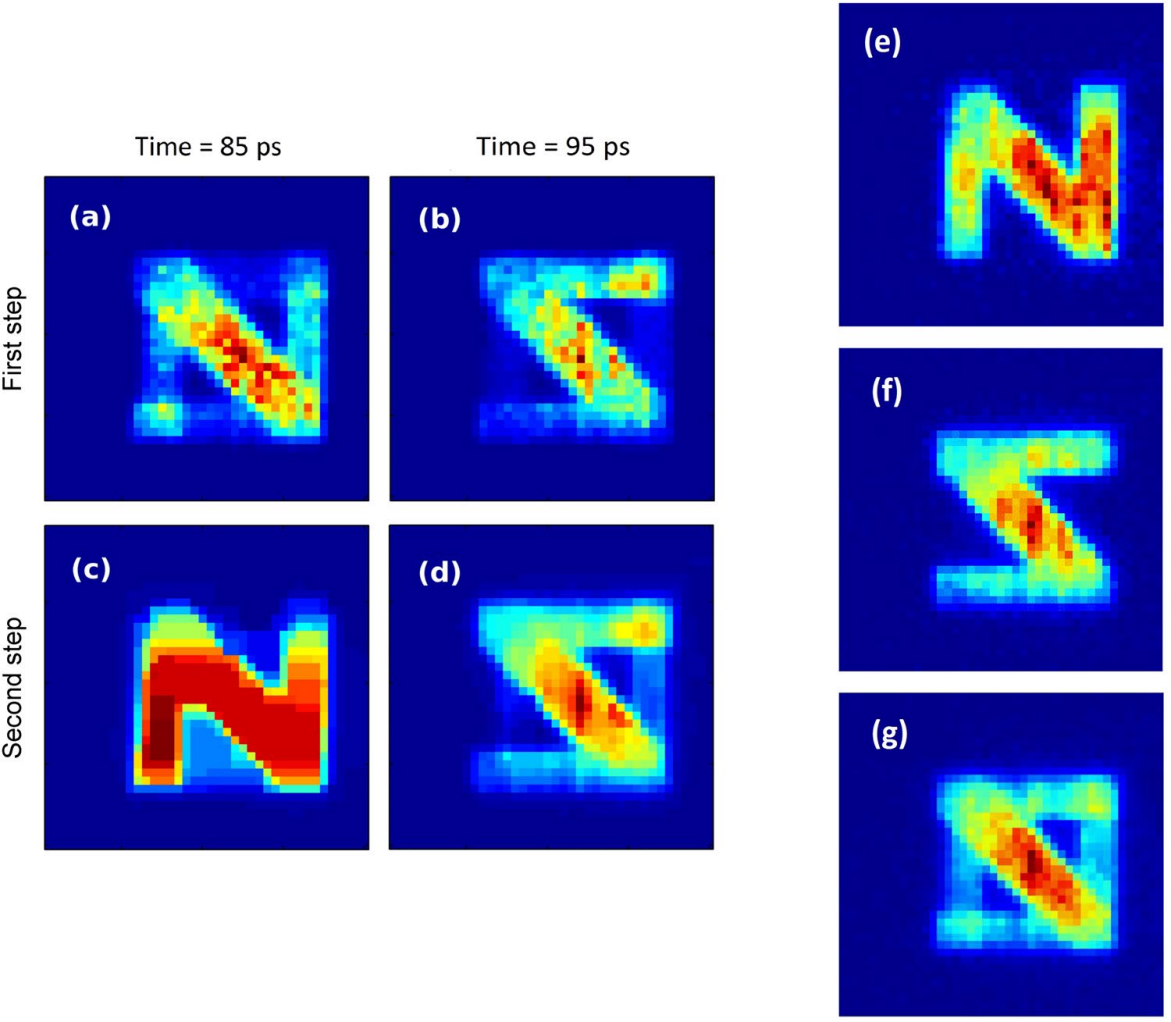
Reconstructed images after the first (**a**,**b**) and second (**c**,**d**) steps of the algorithm, for the 10-ps delay deconvolved data shown in (Fig. 2). All figures are normalized to their own relative maximum value. The second step of the algorithm is used to refine the image by running a new deconvolution process which uses the peak’s position, given by the first step, and employs a different regularization term (total variation) in order to promote smoothness. For an easier comparison, we show the single frames relative to (**e**), the “N” arm alone, in (**f**) the “mirrored-Z” arm alone and in (**g**) both arms.

Finally, we compare the first and second step of our method in Fig. 5. As previously discussed, once the first step of our algorithm has located the peak positions, we can take advantage of eventual spatial correlations between pixels in order to improve the image appearance. To do so, a second step is built in where piece-wise constant intensity profiles are promoted thanks to the total variation regularization in the deconvolution process. In this way, we obtain the overall smoother images reported in Fig. 5. In these figures, there are still residual errors in the discrimination of the two patterns due to short delay between the pulses (10 ps). Such errors decrease as we increase the delay pulses.

### Data availability

All relevant data present in this publication can be accessed at 10.17861/2408c175-89d1-42d9-9616-be9cf6b234cf.

## Conclusion

We have introduced a novel algorithm for improving the temporal deconvolution of a sequence of images subject to temporal blurring (due e.g. to time-gated detection with gates much longer than the image separation) and corrupted by noise. This algorithm is general in that, while it relies on a large number of spatially distributed pixels with similar temporal information, it does not require spatial correlation between the pixels. Furthermore, we do not make assumptions on the shape or number of pulses beyond a basic assumption of sparsity. We demonstrate experimentally the validity of our method by reconstructing two images encoded in two femtosecond pulses overlapped in space and separated in time by 10 ps, imaged with an ICCD camera using a 1000-ps gate, stepped in 10-ps increments. Compared with the results for a single-pixel algorithm, we observe an improvement by a factor of four in the temporal resolution. Moreover, we investigate the role of the step size showing that it may limit resolution. Since our method relies only on the availability of a large number of pixels, the results reported here are general and versatile, and in principle may be applied to most time-resolved imaging processes under the assumption of sparse signals in the temporal domain. Therefore, possible applications might be found in the free space telecommunication field^10,15,16^ and for time gated lidar imaging^17–19^.
